# Supplementary material for: Rhamnose Binding Protein as an Anti-Bacterial Agent—Targeting Biofilm of Pseudomonas aeruginosa
Source: Mar Drugs. 2019 Jun 14;17(6):355. doi: 10.3390/md17060355 (PMC6628293; doi:10.3390/md17060355)
Supplement: Supplementary file 1 [file marinedrugs-17-00355-s001.zip › Supplymantary Information20190524.docx]

Supporting Information

**Table S1** FPLC purification scheme of rHPL_OE_

|  | Starting volume | Primed solution | Flow rate |
| --- | --- | --- | --- |
| Binding | 0 mL | Sample in resuspension buffer | 2 mL/min |
| Wash I | 75 mL | 95% Binding Buffer  5% Elution Buffer | 3 mL/min |
| Wash II | 135 mL | 90% Binding Buffer  10% Elution Buffer | 3 mL/min |
| Wash III | 210 mL | 85% Binding Buffer  15% Elution Buffer | 3 mL/min |
| Wash IV | 285 mL | 70% Binding Buffer  30% Elution Buffer | 3 mL/min |
| Elution | 360 mL | 60% Binding Buffer  40% Elution Buffer | 3 mL/min |
| Equilibrium | 510 mL | Binding Buffer | 3 mL/min |
| Method end | 560 L |  |  |

*Column: HisTrap^™^ HP immobilized metal ion affinity chromatography

*Resuspension buffer: 20 mM Tris, 200 mM NaCl, pH 7.2

*Binding buffer: 20 mM Tris, 200 mM NaCl, pH 7.2

*Elution buffer: 20 mM Tris, 200 mM NaCl, 2 M imidazole, pH7.2


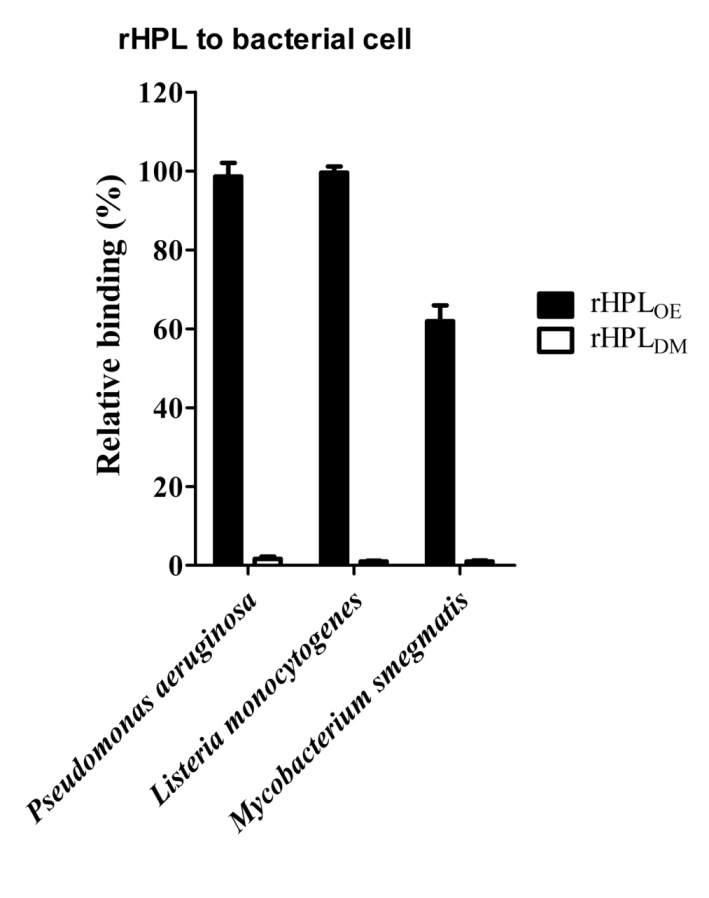


(A)

**
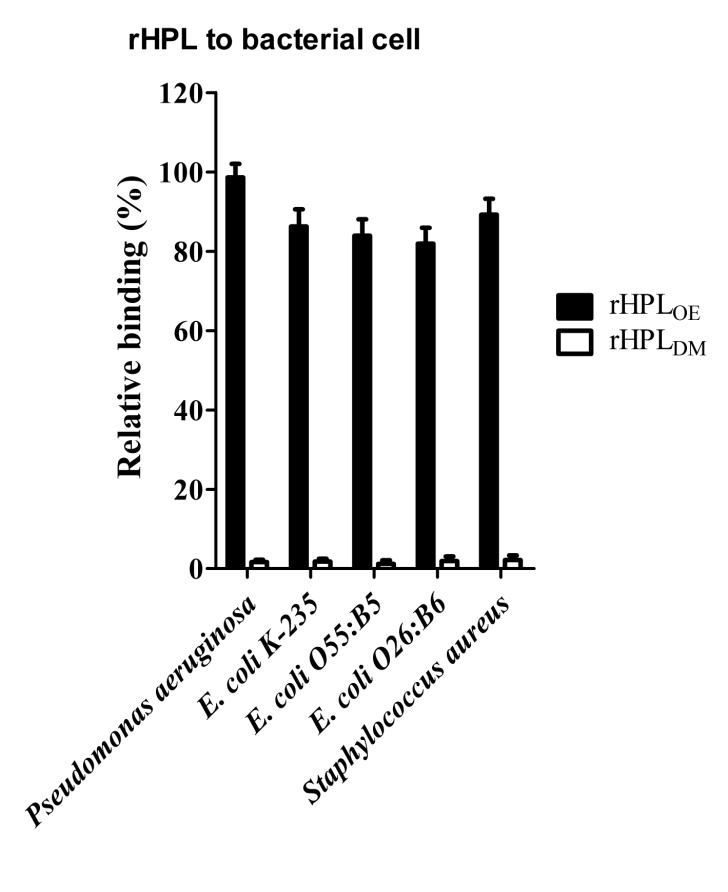
**

(B)

**Figure S1.** Bacterial cell or PAMP binding activity of rHPL_OE_ and rHPL_DM._ Bacterial cell (A) or PAMP (B) binding activity of rHPL_OE_ or rHPL_DM_ was determined by direct ELISA. 1 μM rHPL_OE_ or rHPL_DM_ was applied to 96-well plate coated with bacterial cell (5 × 10^7^ cells each well) or PAMP (0.5 μg each well). PBS buffer was applied as blank. Anti-His monoclonal antibody and anti-mouse IgG polyclonal antibody conjugated HRP was used to detect the binding. Each value was the average of three measurements where presented data was mean ± SD. All means were compared by One-way ANOVA. ***P<0.001 versus the rHPL_OE_ group.


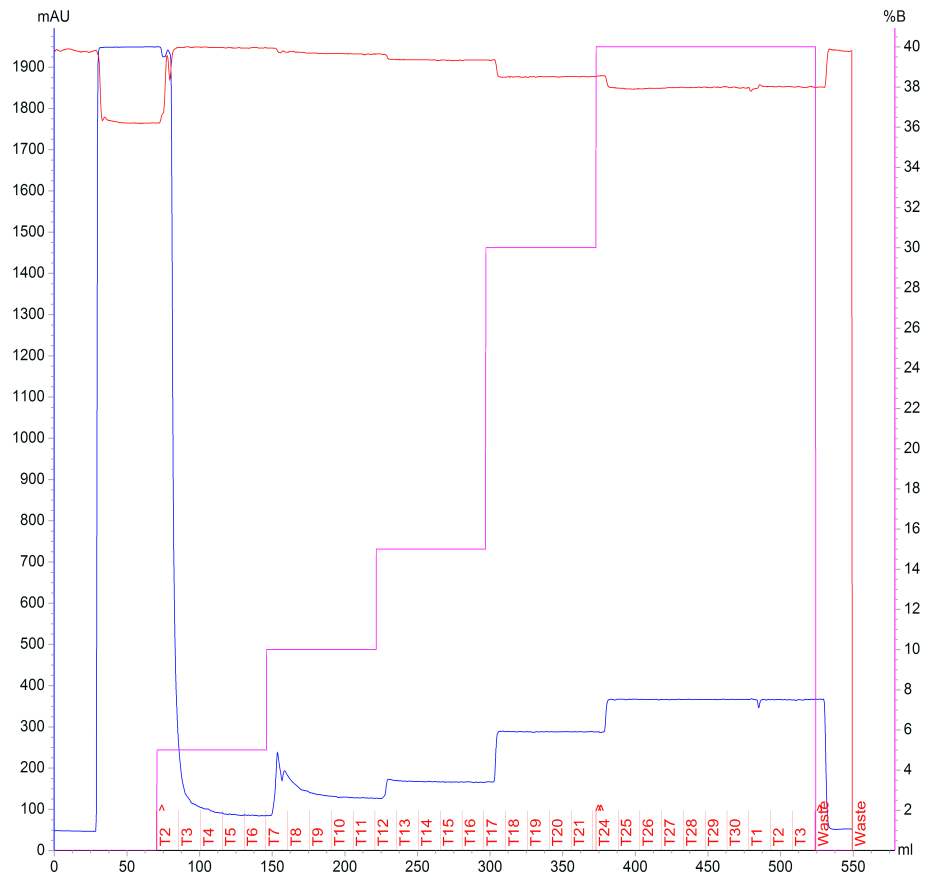


(A)


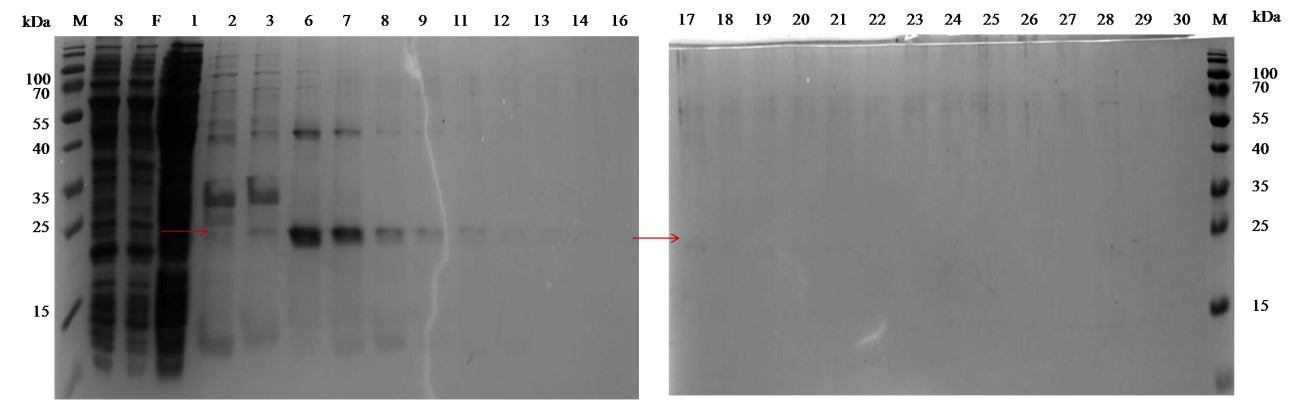


(B)

**Figure S2.** Purification and characterization of rHPL_OE_ with HisTrap™ HP immobilized metal ion affinity chromatography. (A) Chromatographic profile of rHPL_OE_ purification. 90 milliliters of *E. coli*cell lysate containing induced rHPL_OE_ was loaded onto nickel column and separated with a gradient of imidazole (purple trace) at a flow rate of 2 mL per minute at 26 °C and UV absorbance at 280 nm (blue trace) was monitored by ÄKTA^™^ start chromatography systems. Fractions were automatically collected every 15 mL (T2 to T33 fraction No. 1 to fraction number No. 32 (B) Purification efficiency analysis of rHPL_OE_. Collected fractions were analyzed by 15% (w/v) SDS-PAGE. The molecular weight of rHPL was approximately 19 kDa. Lane M: molecular weight marker; Lane S: soluble protein extracts in supernatant from total *E. coli* lysate; Lane F: flow-through of unbound supernatant; Lanes 1 to 30: fraction number (fraction number 2 to 5 were collected as “wash”, fraction number 6 to 30 were collected as “eluent”).


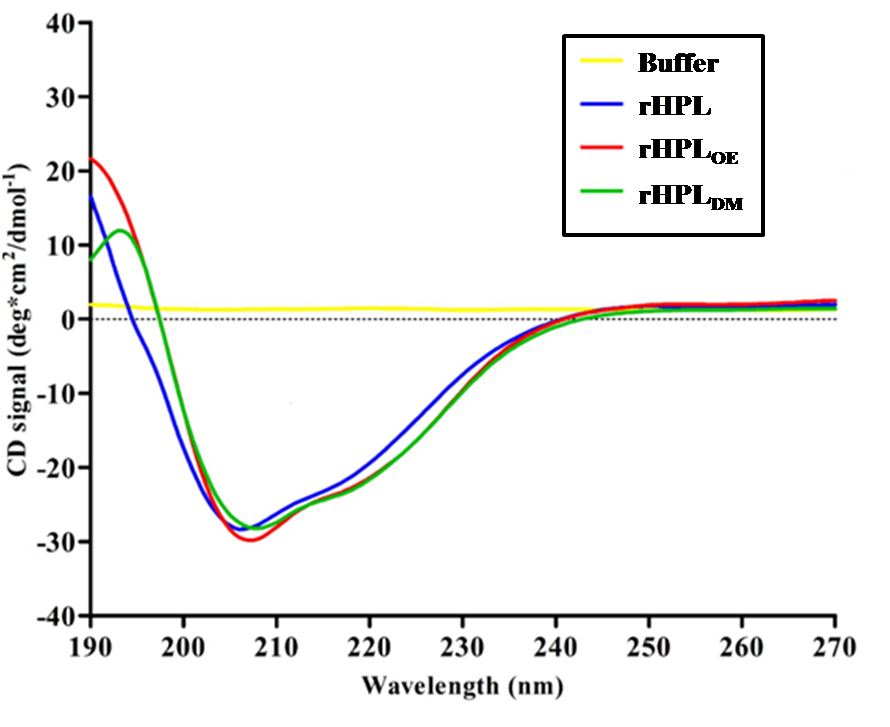


**Figure S3.** Secondary structure analysis of rHPL_OE_, rHPL_DM_ and rHPL using circular dichroism (CD). The CD spectrum of 50 μM rHPL_OE_, rHPL_DM_ or rHPL in 20 mM sodium phosphate (pH 7.2) was determined at 25 ºC. CD spectra were recorded on an Aviv CD spectrometer (model202) equipped with a 450-W xenon arc lamp. Far-UV spectral analysis at 190 to 270 nm was performed in a rectangular quartz cuvette with a 0.1 cm path length at 25 °C using a scan rate of4 nm/sand a bandwidth of 0.5 nm. Each spectrum was the average of three consecutive scans and was baseline-corrected by subtracting the spectrum of buffer alone at the same temperature. The values were indicated as the mean and smoothed with 10 of neighbors on each size to average.

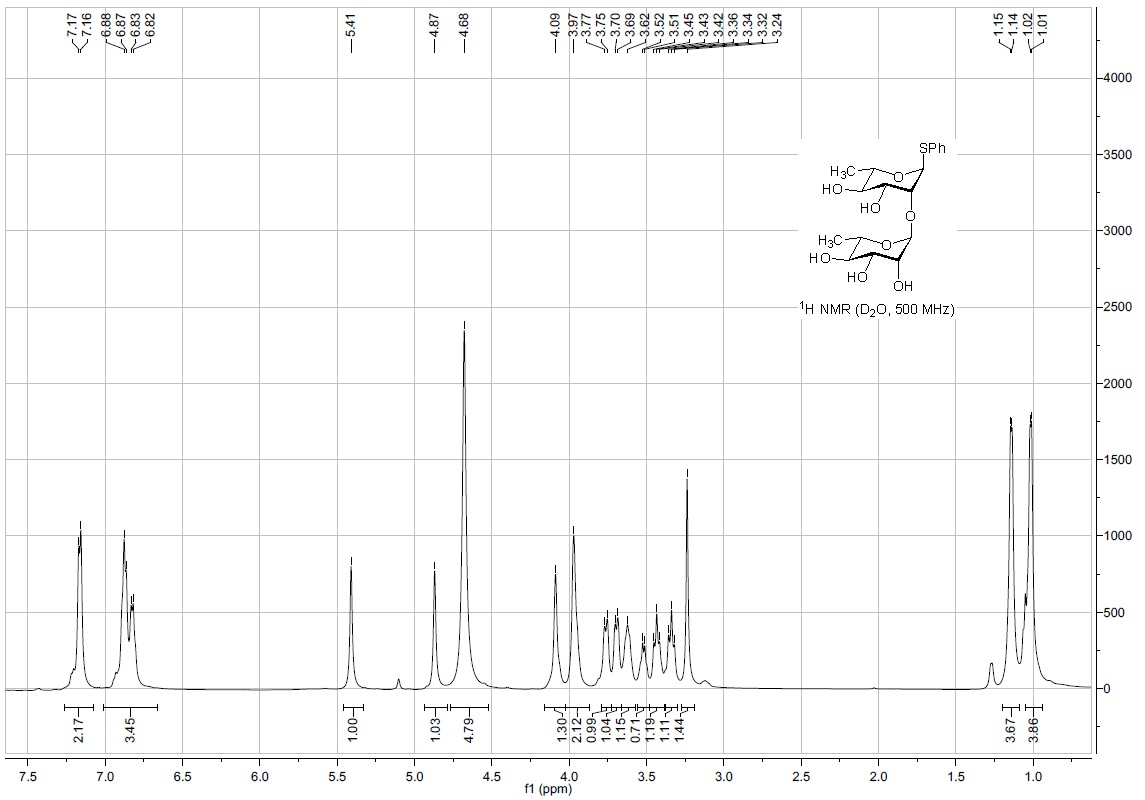


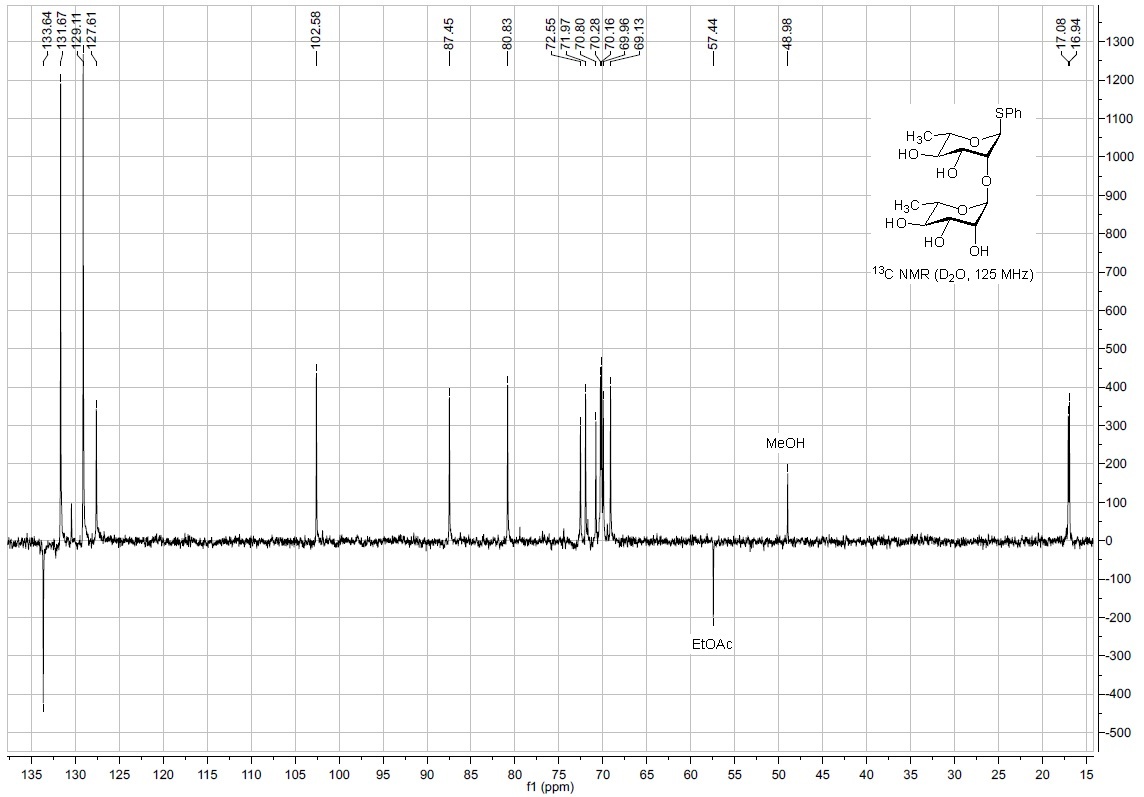


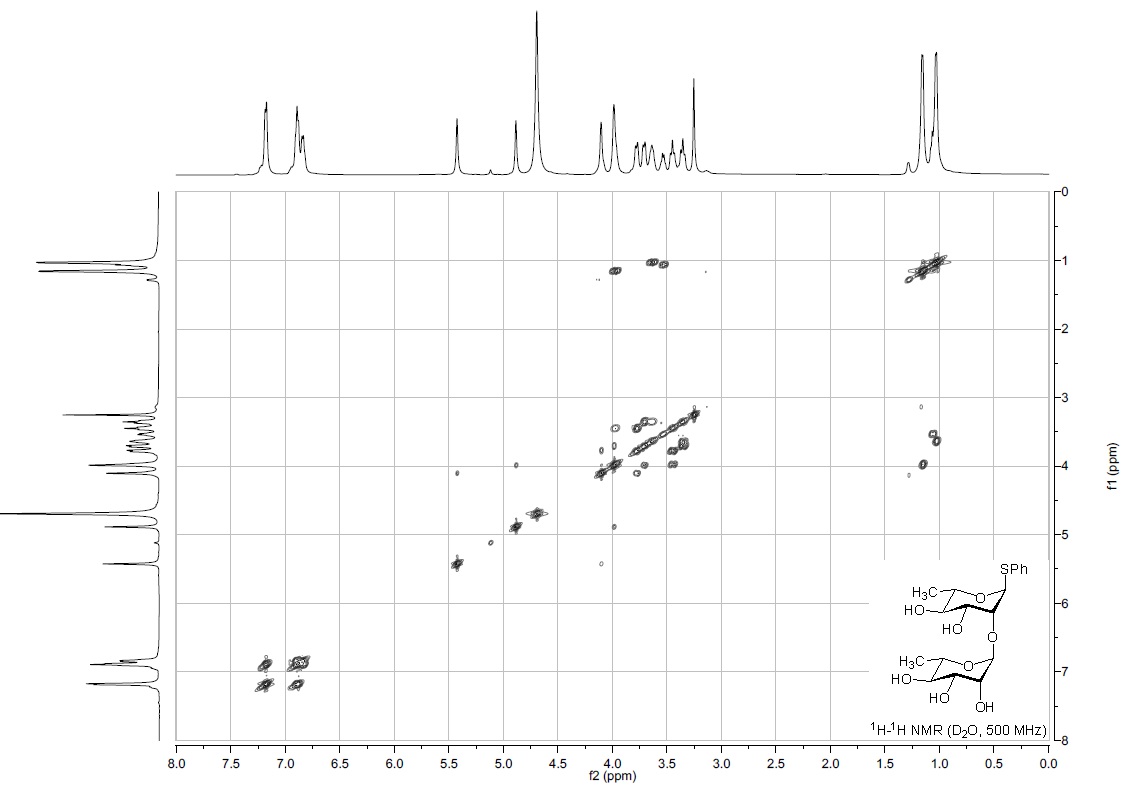


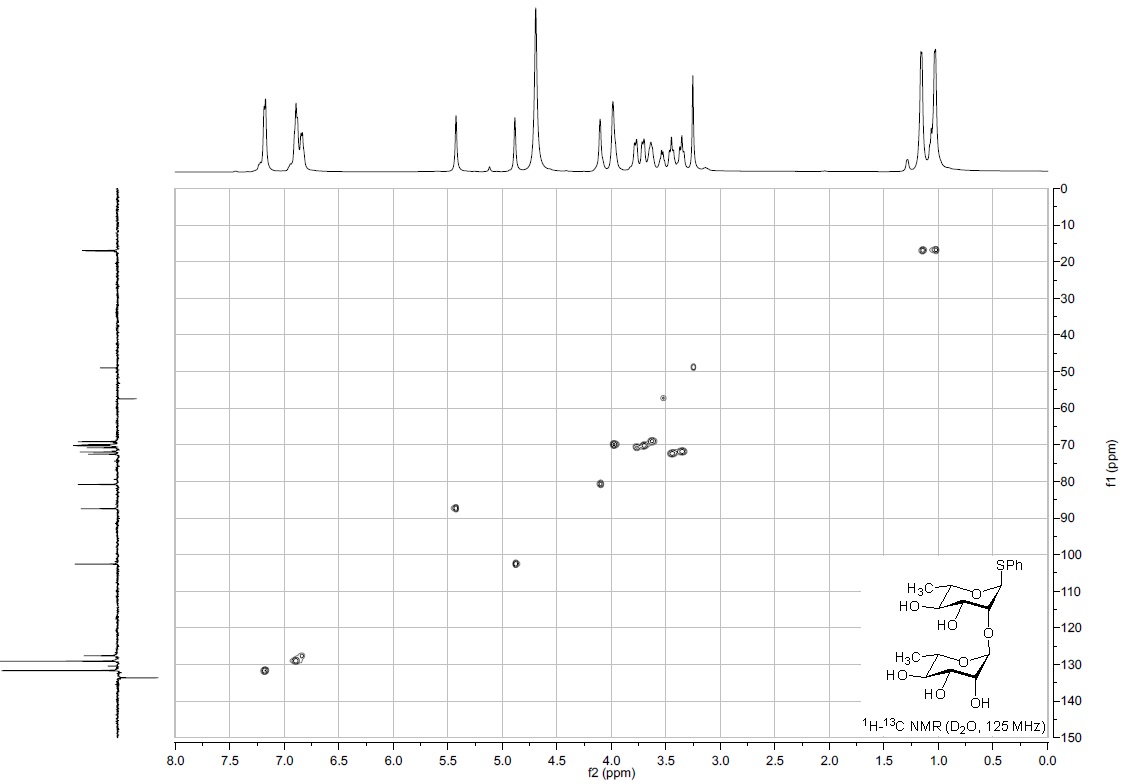


**Figure S4.** Structure and NMR spectra (^1^H and ^13^C) of pehnylthio-1-2-rhamnobioside. Phenyl α-l-rhamnopyranosyl-(1→2)-1-thio-α-l-rhamnopyranoside. The title compound was isolated as a colourless syrup. [α]_D_^25^ −169.3 (*c* 0.13, MeOH); *R*_f_ = 0.45 (75:25 CH_2_Cl_2_/MeOH); ^1^H NMR (500 MHz, D_2_O) *δ* 7.17-6.82 (m, 5H, arom), 5.41 (s, 1H, H-1), 4.87 (s, 1H, H-1’), 4.09 (s, 1H, H-2), 3.97 (s, 2H, H-2’, H-5), 3.76 (d, *J* = 8.4 Hz, 1H, H-3), 3.69 (d, *J* = 8.1 Hz, 1H, H-3’), 3.63-3.60 (m, 1H, H-5’), 3.43 (t, *J* = 8.9 Hz, 1H, H-4), 3.34 (t, *J* = 9.1 Hz, 1H, H-4’), 1.14 (d, *J* = 4.1 Hz, 3H, C*H*_3_), 1.02 (d, *J* = 4.0 Hz, 3H, C*H*_3_’) ppm; ^13^C NMR (125 MHz, D_2_O) *δ* 133.6 (1C, C_q_ arom), 131.7, 129.1, 127.6 (5C, arom), 102.6 (1C, C-1’), 87.5 (1C, C-1), 80.8 (1C, C-2), 72.6 (1C, C-4), 72.0 (1C, C-4’), 70.8 (1C, C-3), 70.3 (1C, C-2’), 70.2 (1C, C-3’), 70.0 (1C, C-5), 69.1 (1C, C-5’), 17.1, 16.9 (2C, 2 x *C*H_3_) ppm. MS (UHR ESI-QTOF): *m/z* calcd for C_18_H_26_NaO_8_S: 425.1241 [M+Na]^+^; found: 425.1246.

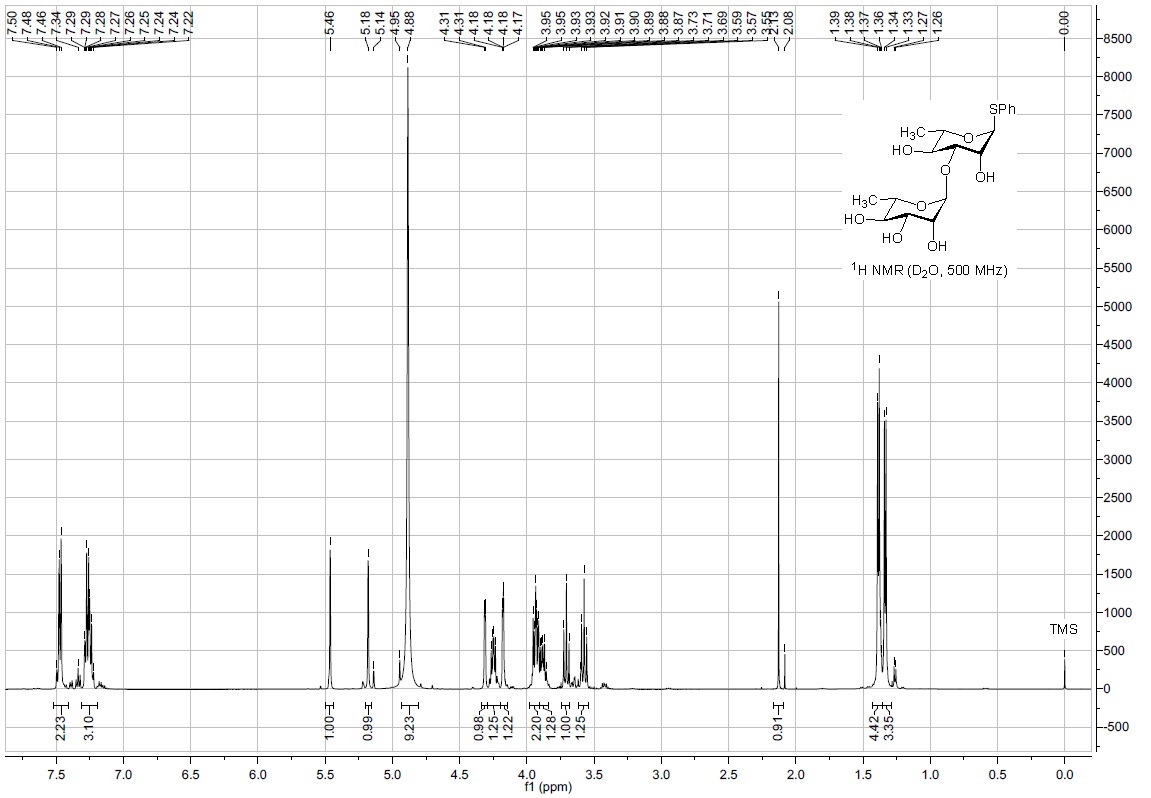


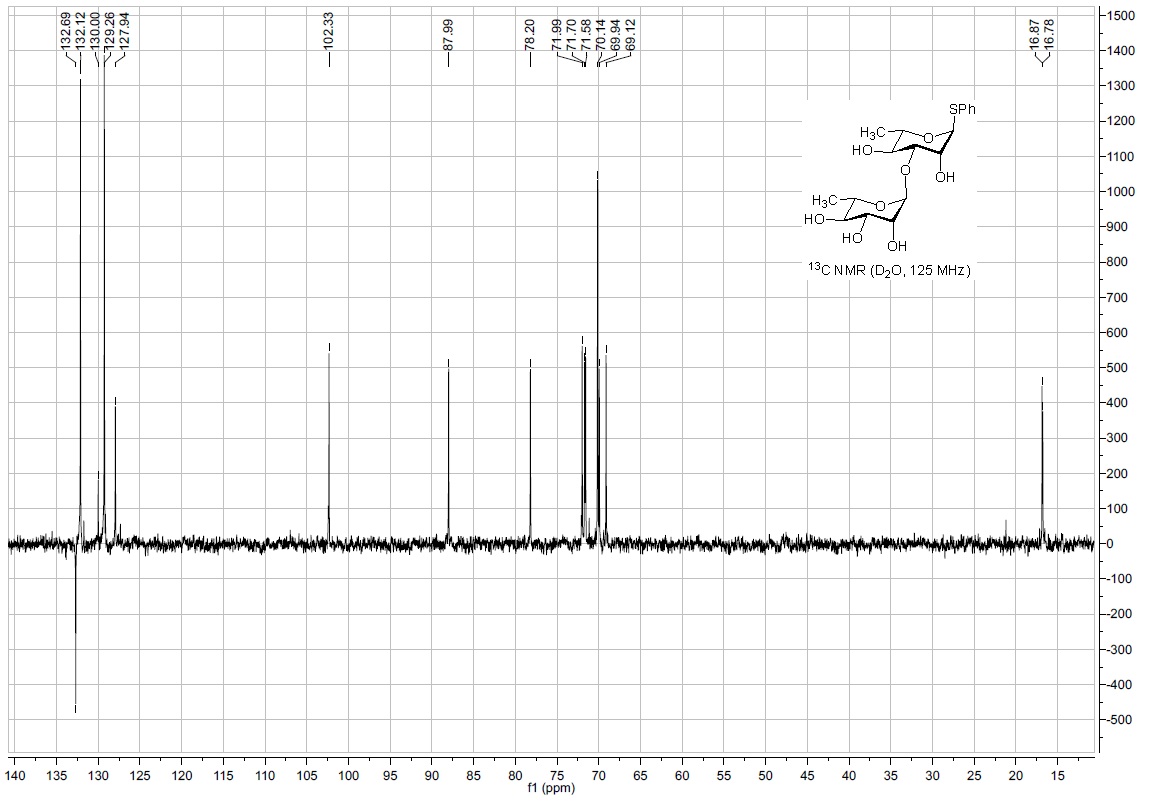


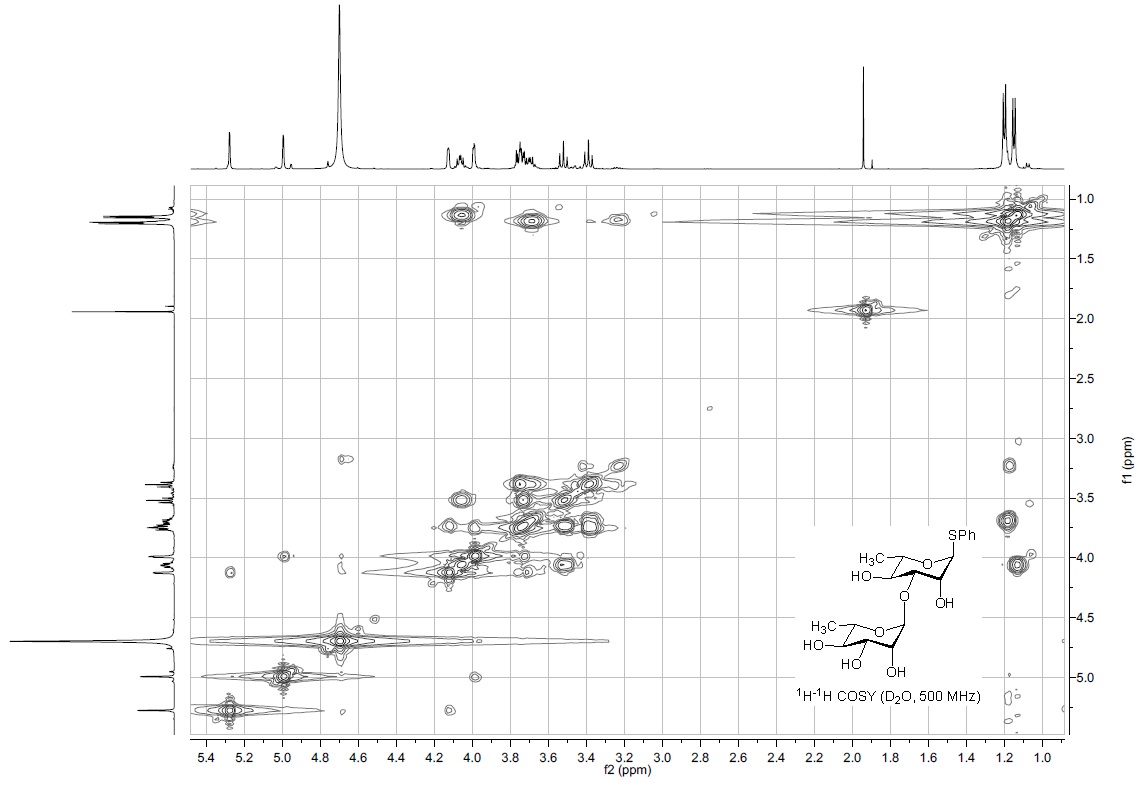


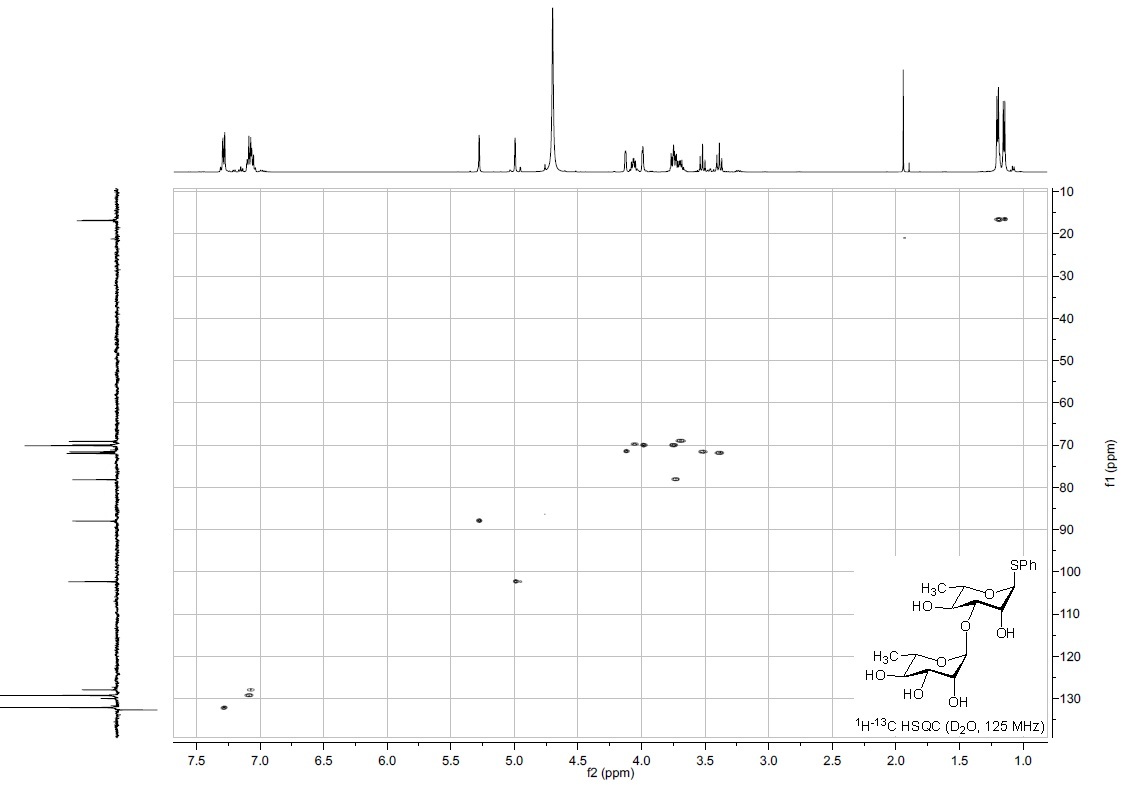


**Figure S5.** Structure and NMR spectra (^1^H and ^13^C) of phenylthio-1-3-rhamnobioside. Phenyl α-l-rhamnopyranosyl-(1→3)-1-thio-α-l-rhamnopyranoside. The title compound was isolated as a colourless syrup. [α]_D_^25^ −165.0 (c 0.14, MeOH); Rf = 0.54 (75:25 CH_2_Cl_2_/MeOH); ^1^H NMR (500 MHz, D2O) δ 7.50-7.22 (m, 5H, arom), 5.46 (s, 1H, H-1), 5.18 (s, 1H, H-1’), 4.31 (dd, *J* = 1.4 Hz, *J* = 2.7 Hz, 1H, H-2), 4.25 (dq *J* = 6.2 Hz, *J* = 12.4 Hz, 1H, H-5’), 4.18 (dd, *J* = 1.5 Hz, *J* = 3.1 Hz, 1H, H-2’), 3.95-3.91 (m, 2H, H-3, H-3’), 3.90-3.86 (m, 1H, H-5), 3.71 (t, *J* = 9.6 Hz, 1H, H-4’), 3.57 (t, *J* = 9.6 Hz, 1H, H-4), 1.39 (d, *J* = 6.2 Hz, 3H, CH_3_), 1.34 (d, *J* = 6.2 Hz, 3H, CH_3_’) ppm; ^13^C NMR (125 MHz, D_2_O) δ 132.7 (1C, Cq arom), 132.1, 129.3, 127.9 (5C, arom), 102.3 (1C, C-1’), 88.0 (1C, C-1), 78.2 (1C, C-3), 72.0 (1C, C-4), 71.7 (1C, C-4’), 71.6 (1C, C-2), 70.1 (2C, C-2’, C-3’), 69.9 (1C, C-5’), 69.1 (1C, C-5), 16.9, 16.8 (2C, 2 x CH_3_) ppm; MS (UHR ESI-QTOF): m/z calcd for C_18_H_26_NaO_8_S: 425.1241 [M+Na]+; found: 425.1244.

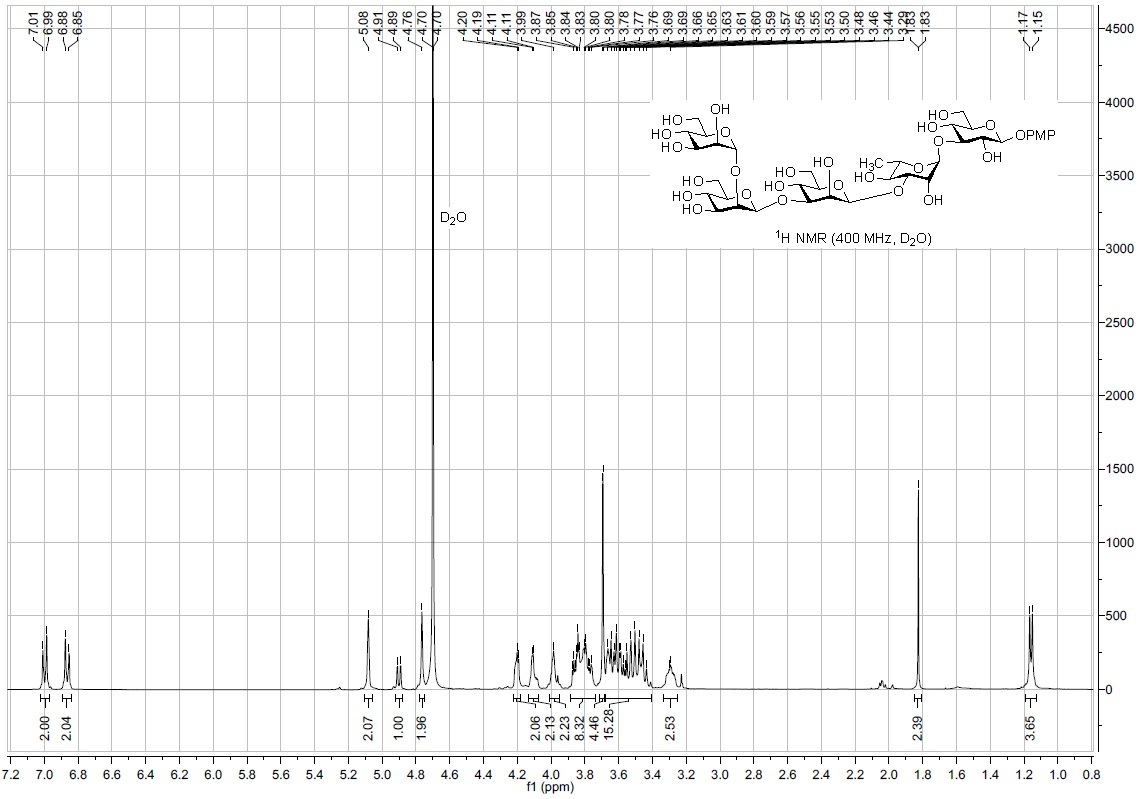


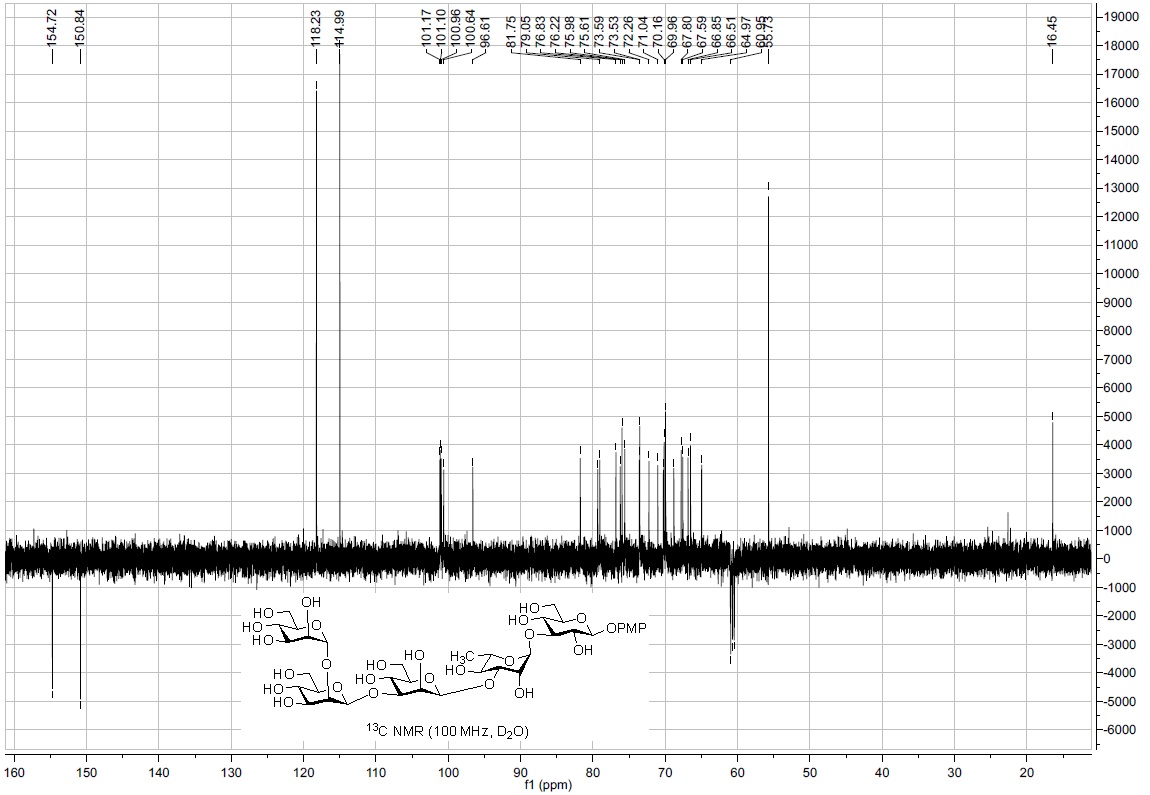


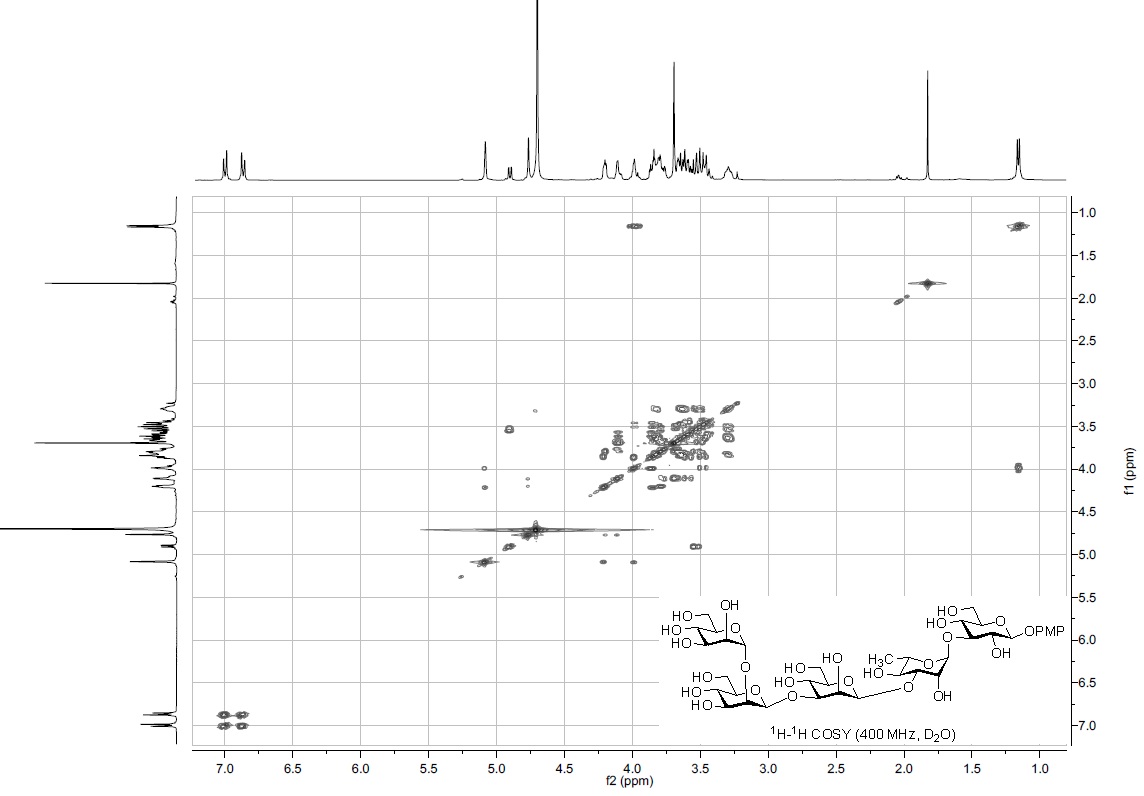


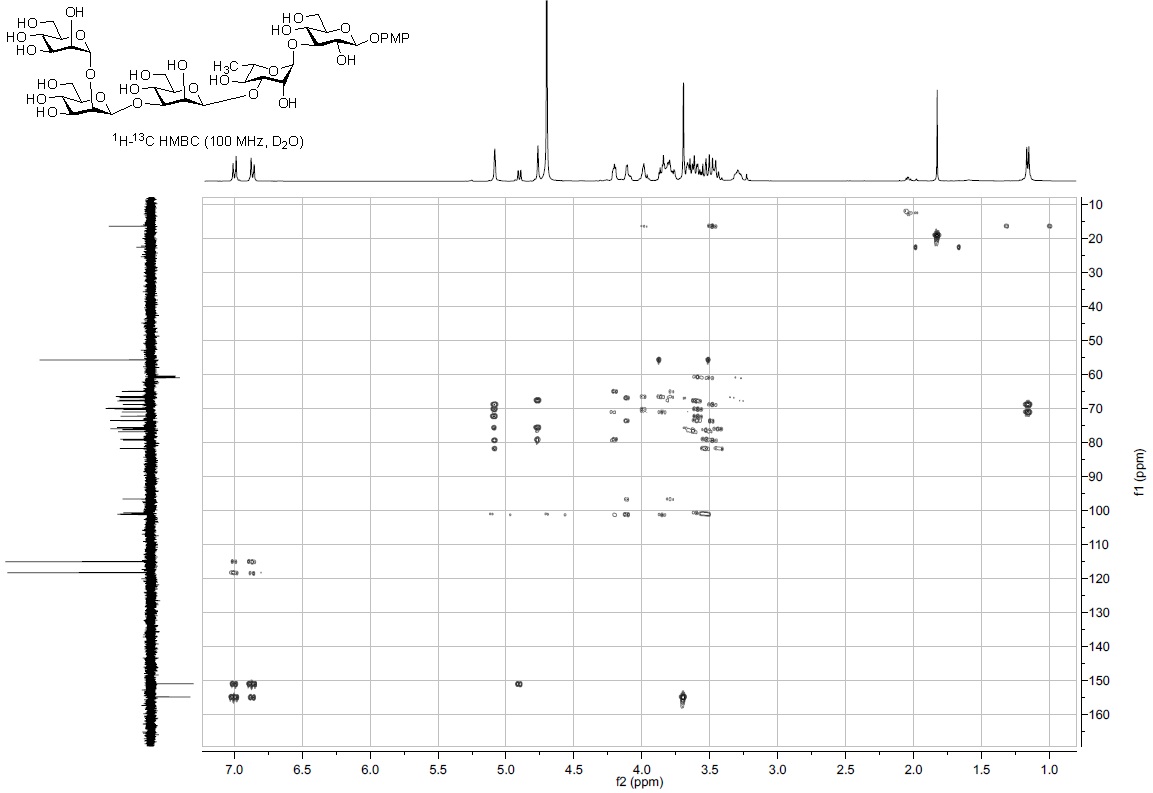


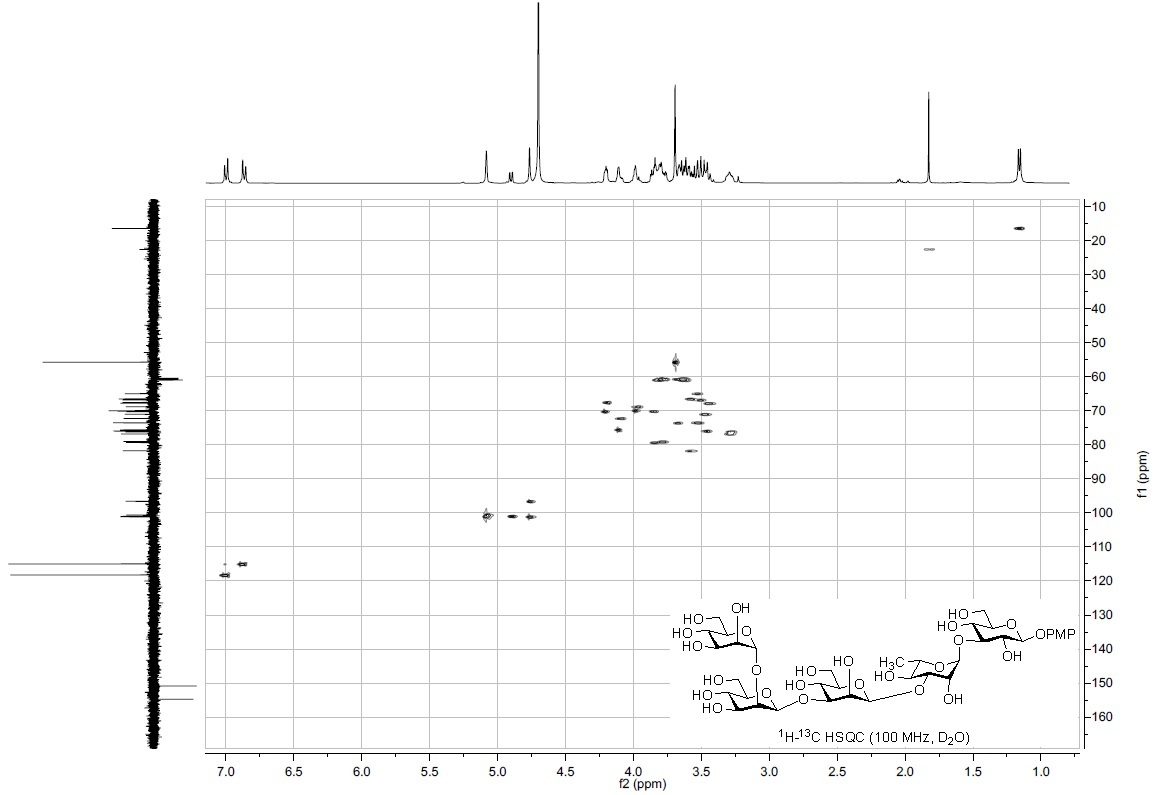


**Figure S6.** Structure and NMR spectra (^1^H and ^13^C) of Psl-pentasaccharide. 4-Methoxyphenyl α-d-mannopyranosyl-(1→2)-β-d-mannopyranosyl-(1→3)-β-d-mannopyranosyl-(1→3)-α-l-rhamnopyranosyl-(1→3)-β-d-glucopyranoside. The title compound was isolated as a white solid. [α]_D_25 −13.5 (c 0.14, MeOH); Rf = 0.29 (7:6:1 CH_2_Cl_2_/MeOH/H_2_O); 1H NMR (400 MHz, D2O) δ 7.01-6.85 (m, 4H, arom), 5.08 (s, 2H, H-1-II., H-1-V.), 4.90 (d, J1,2 = 7.7 Hz, 1H, H-1-I.), 4.76 (s, 2H, H-1-III., H-1-IV.), 4.21-4.19 (m, 2H, H-2-III., H-2-V.), 4.11-4.08 (m, 2H, H-2-IV., H-5-V.), 3.99-3.96 (m, 2H, H-2-II., H-5-II.), 3.87-3.83 (m, 2H, H-3-V., H-3-II.), 3.80-3.76 (m, 5H, H-3-III., 4 x H-6a), 3.69 (s, 3H, OCH3), 3.66-3.44 (m, 13H, H-2-I., H-3-I., H-4-I., H-5-I., H-4-II., H-4-III., H-3-IV., H-4-IV., H-4-V., 4 x H-6b), 3.32-3.27 (m, 2H, H-5-III., H-5-IV.), 1.16 (d, *J* = 6.2 Hz, 3H, CH_3_) ppm; 13C NMR (100 MHz, D2O) δ 154.7, 150.8 (2C, 2 x Cq arom), 118.2, 115.0 (4C, arom), 101.2 (1C, C-1-III.), 101.1 (1C, C-1-V.), 101.0 (1C, C-1-I.), 100.6 (1C, C-1-II.), 96.6 (1C, C-1-IV.), 81.8 (1C, C-3-I.), 79.4 (1C, C-3-V.), 79.1 (1C, C-3-III.), 76.8 (1C, C-5-III.), 76.2 (1C, C-5-IV.), 76.0 (1C, C-5-I.), 75.6 (1C, C-2-IV.), 73.6 (1C, C-3-IV.), 73.5 (1C, C-2-I.), 72.3 (1C, C-5-V.), 71.0 (1C, C-4-V.), 70.3 (1C, C-2-V.), 70.2 (1C, C-3-III.), 70.0 (1C, C-2-II.), 68.8 (1C, C-5-II.), 67.8 (1C, C-4-I.), 67.6 (1C, C-2-III.), 66.8 (1C, C-4-IV.), 66.5 (1C, C-4-II.), 65.0 (1C, C-4-III.), 61.0, 60.9, 60.7, 60.5 (4C, 4 x C-6), 55.7 (1C, OCH_3_), 16.5 (1C, 1 x CH_3_) ppm; MS (UHR ESI-QTOF): m/z calcd for C_37_H_58_NaO_26_: 941.3109 [M+Na]+; Found: 941.3112.


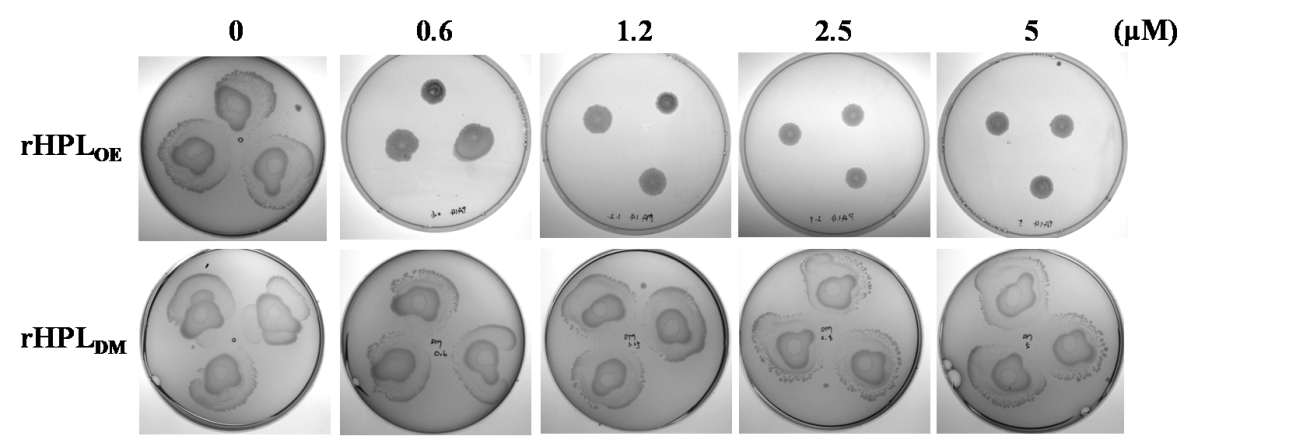


(A)


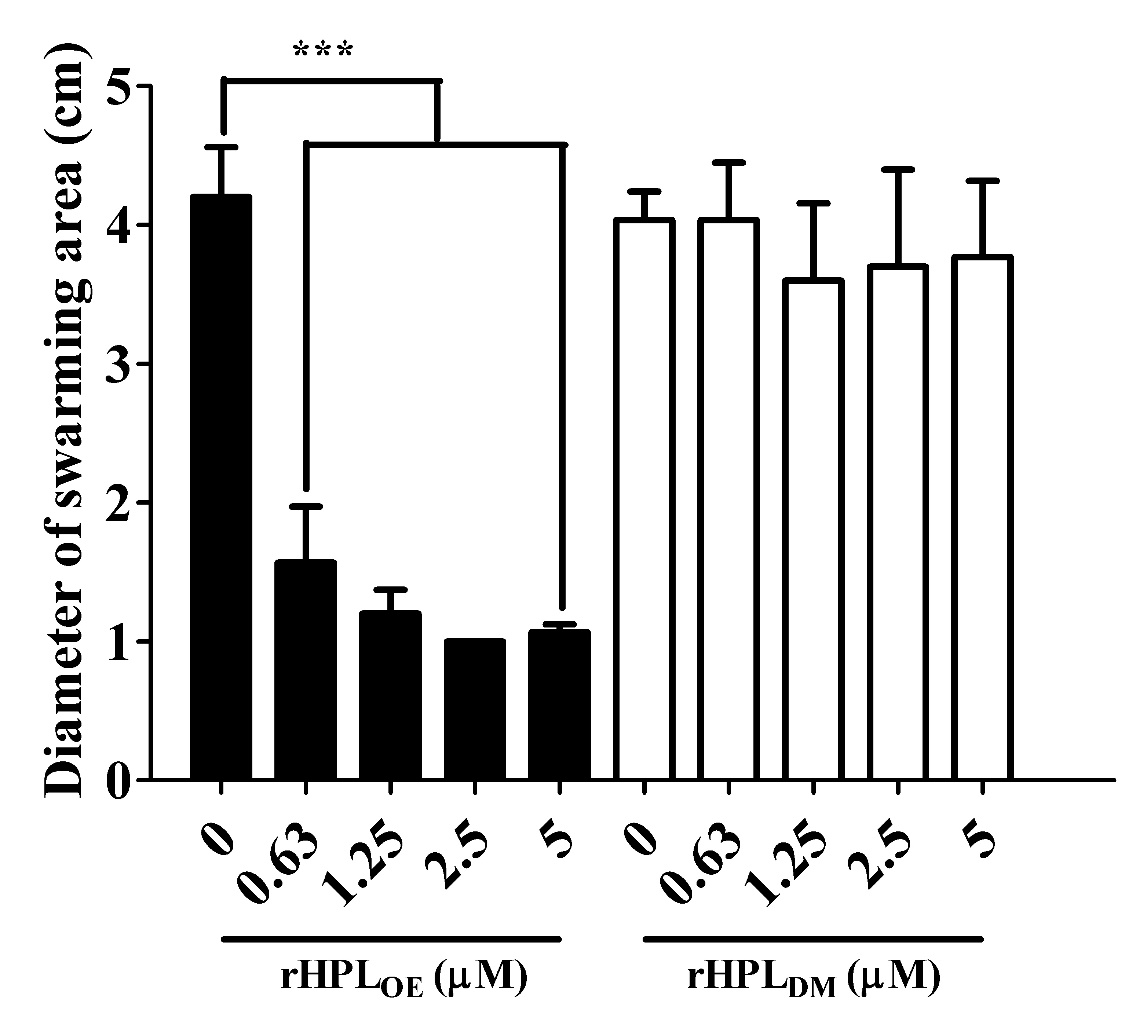


(B)

**Figure S7.** Inhibitory effect on the swarming motility of *P. aeruginosa* PA14 by rHPL_OE_ or rHPL_DM_. The swarming area of *P. aeruginosa* PA14 treated with different concentrations of (A) rHPL_OE_ or rHPL_DM_. The diameter of the swarming area of PA14 treated with different concentrations of rHPL_OE_ or rHPL_DM_ was measured after 72 h (B). Each value was the average of three measurements, where the presented data was the mean ± SD. All means were compared by one-way ANOVA. ****P*<0.001 versus the buffer-treated group.


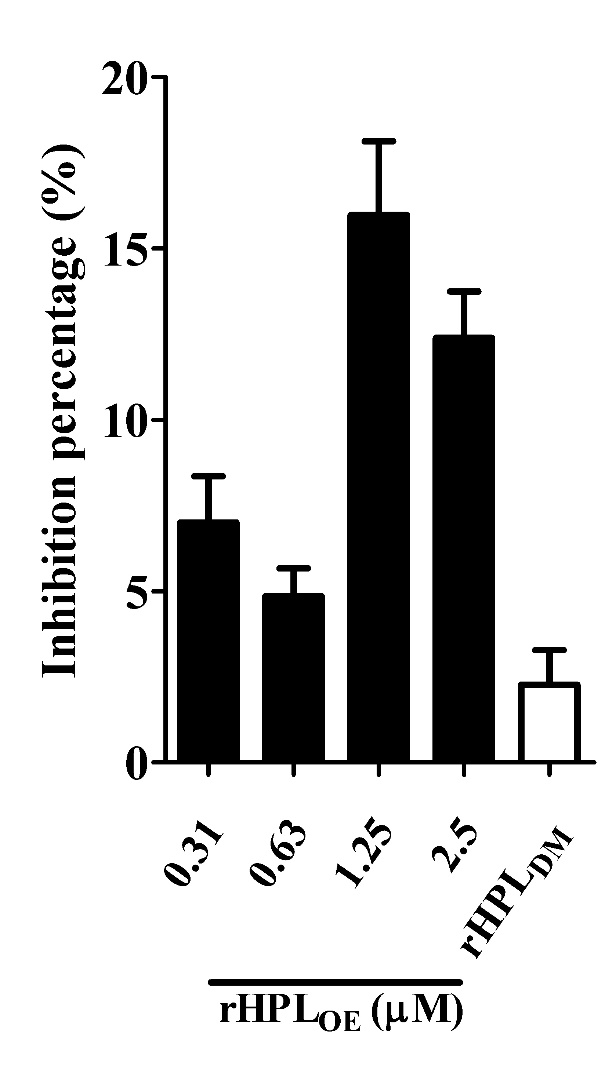


(A)


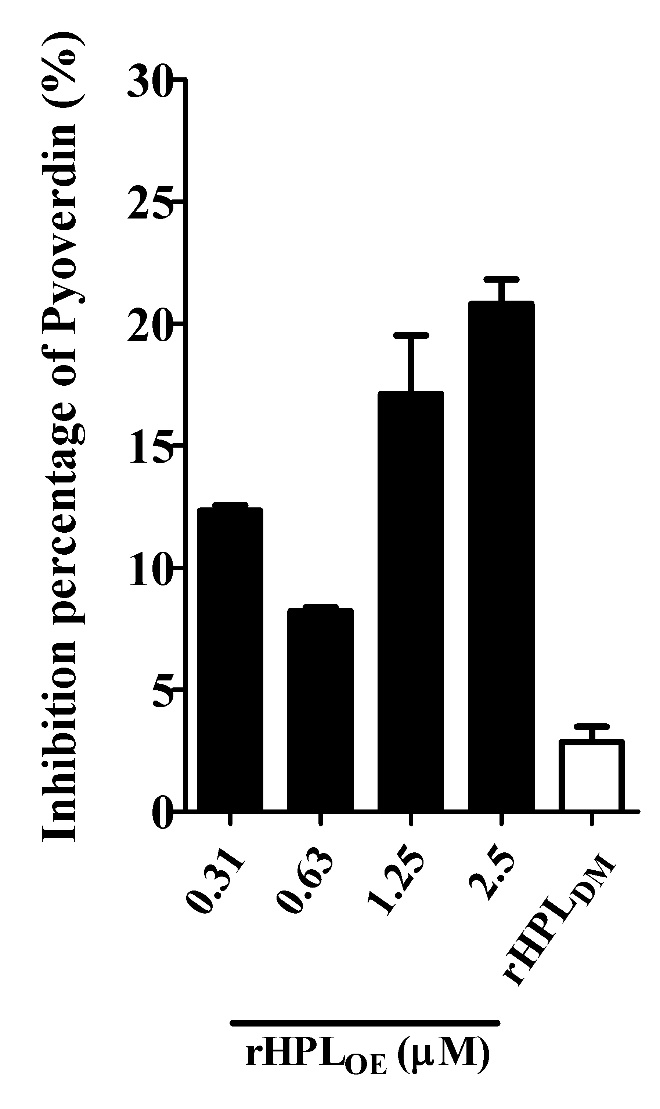


(B)


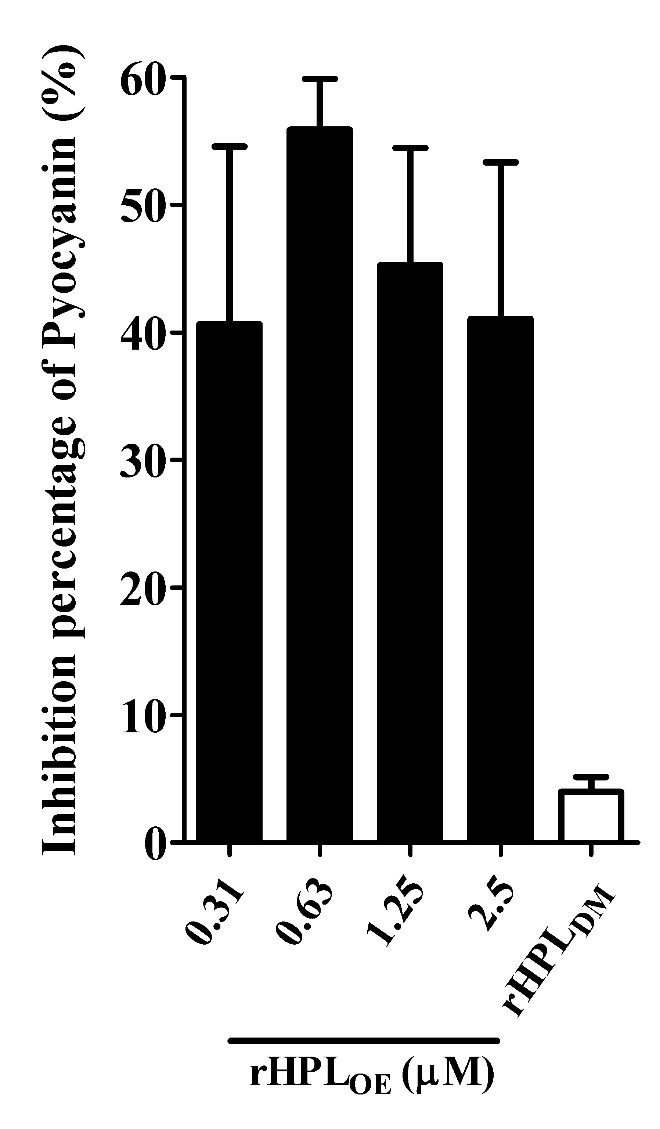


(C)

**Figure S8.** Down-regulation effect on the extracellular protease activities or QS-factors of *P. aeruginosa* PA14 by rHPL_OE_. (A) Extracellular protease activities of *P. aeruginosa* PA14 were measured by the azocasein degradation assay. (B) Secreted pyoverdine was detected by a fluorescence spectrophotometer using 405 nm as the excitation wavelength and 465 nm as the emission wavelength. (C) Secreted pyocyanin was extracted by chloroform and detected by a fluorescence spectrophotometer at 520 nm. The value of the buffer-treated group was set as 0%. All values were expressed as the percentage inhibition with respect to the buffer-treated control. Each value was the average of a triplicate assay, where the presented data was the mean ± SD. **P*<0.05, ***P*< 0.01, ****P*<0.001 versus the buffer-treated group.
